# Real-Time Impedance Monitoring of Epithelial Cultures with Inkjet-Printed Interdigitated-Electrode Sensors

**DOI:** 10.3390/s20195711

**Published:** 2020-10-08

**Authors:** Dahiana Mojena-Medina, Moritz Hubl, Manuel Bäuscher, José Luis Jorcano, Ha-Duong Ngo, Pablo Acedo

**Affiliations:** 1 Department of Electronics Technology, Universidad Carlos III de Madrid, 28911 Madrid, Spain; pag@ing.uc3m.es; 2 Department of Microsystems Engineering, University of Applied Sciences, 10318 Berlin, Germany; moritz.hubl@htw-berlin.de (M.H.); ngo@htw-berlin.de (H.-D.N.); 3 Fraunhofer Institute of Reliability and Micro Integration, 13355 Berlin, Germany; manuel.baeuscher@izm.fraunhofer.de; 4 Department of Bioengineering and Aerospace Engineering, Universidad Carlos III de Madrid, 28911 Madrid, Spain; jjorcano@ing.uc3m.es

**Keywords:** electrochemical biosensor, impedance spectroscopy, inkjet printing, interdigitated electrodes, wound healing monitoring, in vitro skin monitoring

## Abstract

From electronic devices to large-area electronics, from individual cells to skin substitutes, printing techniques are providing compelling applications in wide-ranging fields. Research has thus fueled the vision of a hybrid, printing platform to fabricate sensors/electronics and living engineered tissues simultaneously. Following this interest, we have fabricated interdigitated-electrode sensors (IDEs) by inkjet printing to monitor epithelial cell cultures. We have fabricated IDEs using flexible substrates with silver nanoparticles as a conductive element and SU-8 as the passivation layer. Our sensors are cytocompatible, have a topography that simulates microgrooves of 300 µm width and ~4 µm depth, and can be reused for cellular studies without detrimental in the electrical performance. To test the inkjet-printed sensors and demonstrate their potential use for monitoring laboratory-growth skin tissues, we have developed a real-time system and monitored label-free proliferation, migration, and detachment of keratinocytes by impedance spectroscopy. We have found that variations in the impedance correlate linearly to cell densities initially seeded and that the main component influencing the total impedance is the isolated effect of the cell membranes. Results obtained show that impedance can track cellular migration over the surface of the sensors, exhibiting a linear relationship with the standard method of image processing. Our results provide a useful approach for non-destructive in-situ monitoring of processes related to both in vitro epidermal models and wound healing with low-cost ink-jetted sensors. This type of flexible sensor as well as the impedance method are promising for the envisioned hybrid technology of 3D-bioprinted smart skin substitutes with built-in electronics.

## 1. Introduction

From cell-laden hydrogels to semiconductors, printing techniques enable a wide range of functional materials to be patterned onto a variety of substrates from a digital design. The advantages that lie in the low-material consumption/wastage, speediness and accuracy of these revolutionary techniques have not been concealed from the eyes of either electronic or tissue engineers. Such distant scientific fields have thus harnessed this powerful tool for the fabrication of electronic devices and complex biological structures independently. Our lab, for instance, has fabricated dermo-epidermal engineered skin using 3D bioprinting and tested them in vivo as dermis/epidermis substitutes in rat models [1]. We have also exploited screen printing to manufacture biocompatible humidity sensors [2] and developed organic-based actuators with printing-compatible materials to study the mechanical activation of biological processes [3]. Not surprisingly, our research has been gradually fueling the vision of a hybrid platform capable of fabricating these sensors, actuators and living skin tissues simultaneously. Although this concept would unleash an all-printed, ready-to-implant, dermo-epidermal skin substitutes with built-in electronics (able to report both cell´s status and functions and activate regenerative outcomes), as cyborg tissues [4], this endeavor remains challenging.

From the perspective of monitoring tissue-engineered skin models, a non-invasive, non-destructive and real-time method is vital. Current practice for assessing both the fabrication and the eventual transplant of such laboratory-grown tissues relies on immunocytochemical testing and professional surveillance [1,5,6,7] which, unfortunately, often involves not only subjective judgments but also samples’ destruction. In this regard, the impedance-based method appears as an appealing methodology to assess both cells and tissues´ status since it has been widely accepted as a label-free, easy, and non-destructive technique [8,9,10]. Indeed, a body of literature exists demonstrating the potential of impedance-based methods to monitor cell proliferation, adhesion and morphology [11,12,13], quantify biomass in suspensions, and detect bacteria [12]. To manufacture electrodes and, eventually, such impedance-based devices, microfabrication techniques such as wet etching, photolithography and physical metal evaporation have been traditionally adopted on bulky substrates like glass and silicon [9].

Clean-room-fabricated impedance-based sensors have been proved valuable as electrochemical analytical tools. Yet, they have lacked in the contemplation of the mechanical mismatch between the stiff silicon-based electronics and the fragile living cells. Standard electronic materials are several orders of magnitude stiffer than cells, which respond to the mechanical properties of the interfaced surfaces [3,14]. To overcome this major bundle and endow cells softer interfaces, significant effort has been directed towards using flexible low-cost substrates in the manufacturing of the sensing devices, which in turn, has deviated the fabrication methods from the standard clean-room facilities. A very recent example was reported by Tonello et al. who used carbon-based monopolar electrodes patterned on flexible polyimide substrate to monitor (using impedance) mesenchymal stromal cells seeded into scaffolds of gelatin-chitosan [15]. Other studies have shown the potential of printed electronics for the development of sensors mechanically compliant to cells, able to monitor a variety of biomarkers and cell behaviors [16,17,18,19]. However, regarding the fabrication process, previous examples either involve additional clean-room steps or suffer from an incompatibility between the additive electronic manufacturing technique used (e.g., screen printing, aerosol jet printing) and the current bioprinting methods. With an eye toward a future platform to pattern devices and integrate living components concomitantly, a non-contact, gas-free, mask-less, electronic manufacturing technique, compatible with bioprinting, is paramount.

Considering these limitations, inkjet printing technology leverages a promising approach for the fabrication of the sensors. Not only has inkjet printing proved to deposit a wide material portfolio spanning from conductive polymers and dielectric inks to proteins and living cells [20,21], but also it still lies in the foot of its early stage of development, which means innovative applications may be expected to appear within the coming years. Recently, some studies have demonstrated the feasibility of inkjet-printed multielectrode arrays (MEAs) for monitoring cell cultures [22,23,24]. For example, Garma et al. presented a plastic inkjet-printed MEAs to monitor the extracellular potential of cardiac cell cultures [23]. Adly et al. have also shown the potential of inkjet printing for the fabrication of sensors to record extracellular electrical potentials [24,25]. Although those studies have demonstrated the potential of inkjet printing technology as an alternative fabrication to the traditional manufacturing method in the area of bioelectronics, such sensors are most often used for stimulation or recording cellular potential, which for non-electrically excitable epithelial cells remains less relevant. Approaches using inkjet-printed sensors for monitoring cell cultures with other detecting principles are reported less frequently. In this regard, Tonello et al. presented a study using inkjet-printed impedance sensors for monitoring cellular adhesion of myoblast subjected to cyclic stretching [26]. Unfortunately, the authors validated the study using fixed cells and the platform to perform the measurement was incapable of operating in real time. Further effort is still required to accomplish solutions: (1) able to monitor cellular process that are relevant to laboratory-growth skin tissues, (2) able to operate in real-time and long-term, and (3) able to exploit the advantages of inkjet printing as the manufacturing method for the fabrication of flexible and thin sensors. In addition, sensors must operate in a continuous, intimate interface with living cells while allow monitoring in real-time biological processes without any detrimental effect of neither the electrical signal nor the culture maturation.

Following these interests we have ink-jet printed impedance sensors on a flexible polymeric substrate for monitoring a monolayer of a cell line of keratinocytes (HaCaT) in a real-time fashion due to the fact that the major cell that comprises the epidermis is the keratinocyte (90–95%) [27] and HaCaT has been for decades a standard cell model to study the skin tissue [28]. We have considered three relevant cellular processes —proliferation, migration and detachment of keratinocytes— to test the inkjet-printed sensors and demonstrate their potential use in biologically relevant scenarios for laboratory-growth skin tissues. Migration and proliferation of keratinocytes play a key role in the complex processes that are activated and coordinated in the skin tissue during wound healing [29], whereas cell detachment allows us to emulate the homeostatic continuous shedding of dead cells that occur in the outermost cell layer of the stratum corneum once a month [27].

To that aim we have fabricated the sensors, characterized its electrical and topographical properties, and integrated them in 2D cultures as biosensors based on impedimetric transducers. We have investigated the cell-substrate impedance as an indicator of the physiological state of cells with a developed system able to monitor in real-time electrochemical impedance spectroscopy, microscopically observe cell culture and maintain viable cells simultaneously. With this, we support the use of inkjet-printed devices for in vitro impedance monitoring and demonstrates the feasibility of embedding inkjet-printed flexible sensors into a monolayer of cell culture for monitoring the proliferation, migration and detachment of keratinocytes.

## 2. Material and Methods

### 2.1. Fabrication of Interdigitated Sensors

**Figure 1:**
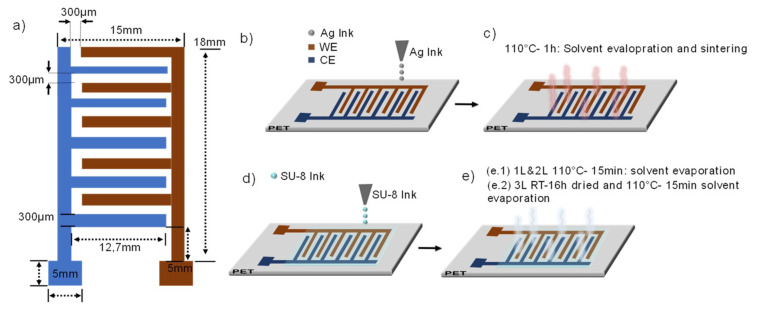
(**a**) Sketch and (**b**–**e**) manufacturing strategies for inkjet printing coplanar capacitors, showing the materials used and curing parameters for the development of the sensors.

Inkjet printing was chosen as the fabrication method due to its mask-less, contactless and compatibility with current bioprinting techniques. The device consists of planar interdigitated electrodes (IDEs) and passive layers. Figure 1a depicts the geometry and dimensions of the IDEs, which were designed to have compatible dimensions with commercially available wells for cell cultures.

The printing process is comprised of two steps. First, a layer of conductive trace was patterned and stacked on the substrate followed by the printing of three layers of a dielectric ink on top of the previously printed conductive lines (Figure 1b–e). The printed conductive ink forms both the working (WE) and counter (CE) electrodes while the dielectric ink provides the required passivation. The printed lines of the active areas were designed to have a width of 300 µm and a gap between them of 300 µm. Conductive lines and the passivation layer were printed with a 15 µm and 20 µm drop spacing, respectively. The sensors were printed under a substrate vacuum, controlling the temperature of both the printing plate and cartridge depending on the ink. The printhead nozzle temperature was set at 28 °C for silver ink and 40 °C for SU-8 ink, while the bed was heated up to a temperature of 45 °C and 31 °C for Ag ink and SU-8, respectively. The printing processes were carried out in a standard laboratory environment in ambient conditions (i.e., neither a filtered enclosure system nor control of temperature or humidity was required). Individual cartridges with nozzles of 10 pL were filled with about 1 mL of the filtered inks (Minisart^®^ syringe filters, average pore size 0.20 μm, Sartorius GmbH, Gotinga, Germany). Without considering the contact pads the sensors´ active area is 195 mm^2^ with 22 IDEs fingers of 12.7 mm length each. The passivation layer was a rectangle of 13 × 14 mm which a nominal area of 182 mm^2^.

A piezoelectric Dimatix Material Printer (DMP-2800TM, Fujifilm-Dimatix, Inc., Santa Clara, CA USA) was employed for inkjet printing the IDEs. Printing patterns were sketched using the GIMP editor program and imported with the Dimatix Bitmap layout software. Polyethylene terephthalate film (PET) with a thickness of 100 µm (PET foils, Mitsubishi Paper Mills, Tokyo, Japan) was selected as a substrate. A silver-nanoparticles ink (AgNP, 796042 Ag, Sigma Aldrich, St. Louis, MI, USA; NBSIJ-MU01, with 15% Ag, ethylene glycol 15–25%, ethanol 1–2%) was employed for the interdigitated electrodes, contact pads and feed lines.

The isolation of the electrodes was achieved using SU-8 ink (MicroChem PriElex^®^ SU-8, MicroChem Corp., Westborough, MA, USA). Both inks show drop-on-demand inkjet compatible specifications and were used as received after filtration. A silver-nanoparticle based ink was chosen for the electrode material since it provides good conductivity and has been recently reported that undergo a spontaneous coalescence process (sintering) at room temperature in presence of chloride ions [30,31]. This self-sintering property opens the possibility of patterning bio components and electronic materials in one single additive platform. However, the cytocompatibility and chemical stability of silver nanoparticles are still not thoroughly understood and has been shown to be highly time- and concentration-dependent [32,33,34]. To generate sensors able to perform impedance recordings in an electrolytic environment, conductive lines were protected using a dielectric-based ink (SU-8) with a twofold objective: firstly as a protection for the electrodes and, secondly, as inert material in direct contact to the cell cultures. The photoresist SU-8 has been previously reported in the literature as biocompatible [35] and it has been recently used as passivation for in vitro electrophysiology due to its transparency [23].

### 2.2. Assembly of IDE-Based Devices and Measuring System

**Figure 2:**
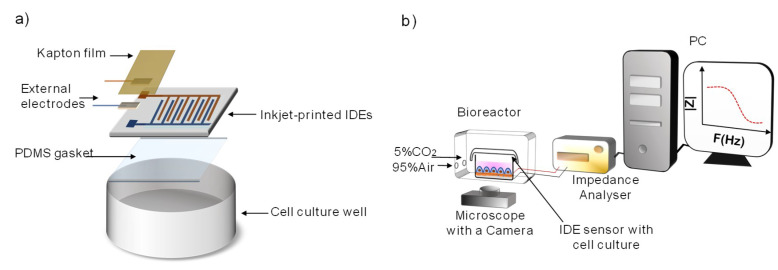
(**a**) Assembly and packaging of the IDE-based devices to perform impedance spectroscopy of cultured cells. (**b**) Schematic representation of the impedance-based biosensor system. The system consists of inkjet-printed IDE sensors, an IDEs-based chamber, an impedance analyzer, a microscope with an integrated bioreactor and a PC to control the images ‘acquisition and the impedance recordings in real-time.

IDEs were custom packaged on an IDEs-based impedance cell culture unit to perform impedance spectroscopy of cultured cells (Figure 2a). To solder external cables on the pads of the IDEs a silver epoxy EPO-TEK^®^H20E (Epoxy Technology, Inc., Billerica, MA, USA) was used. To isolate the exposed welding one-side adhesive Kapton^®^ films were used. The IDEs were glued to cell culture dishes (∅35 mm, ThermoFisher Scientific, Madrid, Spain) by using either a thin layer of polydimethylsiloxane (PDMS, Sylgard^®^ 184, Sigma Aldrich, St. Louis, MI, USA) or a biocompatible silicone grease (Dow Corning™ High-Vacuum Grease, Fisher Scientific, Madrid, Spain). PDMS was prepared in a proportion of 10:1, base and agent, respectively. The silicone grease was used as received in a similar way to the PDMS gasket. To lead the cables out of the Petri dishes, lateral holes were drilled with a 2 mm drill bit (8100 Model, Dremel^®^, Racine, WI, USA) and sealed using hot silicon to prevent any leakage of the culture medium.

The measuring system consists of a commercial impedance analyzer to perform impedance spectroscopy across the inkjet-printed based devices, a microscope with an integrated camera, and the inkjet-printed IDEs-based devices to culture cells (Figure 2b). For microscopy observation, a Leica DMi8 Inverted Microscope (Leica Microsystems CMS GmbH, Wetzlar, Germany) equipped with a CCD camera (Olympus DP26) and its proprietary software LASX Navigator (Leica Microsystems) were used. A bioreactor maintains stable conditions of 5%CO_2_ and 37 °C for cell survival during the prolonged time-lapse/impedance experiments.

### 2.3. Characterization of the Inkjet-Printed Sensors

For the optical evaluation of the sensors a BX53 Digital Fluorescence Microscopy (Olympus Inc., Tokyo, Japan) equipped with a CCD camera (Olympus DP26) and a 10x optical objective was used. The analySIS-getIT software (Olympus^®^, Tokyo, Japan) was used to acquire the images and the ImageJ software (NIH, Bethesda, MD, USA) was employed to quantify the sensor features. The morphology of the printed line was analyzed by using a profilometer (Dektak^XT^ Stylus Profilometer, Bruker, Karlsruhe, Germany). Scanning Electron Microscopy (SEM) was employed to characterize the topography of the printed surfaces. Samples were treated by gold sputtering technique (EM ACE200, Leica Microsystems, Weitzlar, Germany) for 90 sec before imaging.

To electrical (impedance) characterize the inkjet-printed sensors a commercial impedance analyzer (ISX-3 mini, Sciospec Scientific Instruments GmbH, Bennewitz, Germany) and a PC (Sciospec ISX3 v2 software) were used. The excitation signal was sinusoidal with 25 mV of amplitude and a frequency range of 10 Hz to 500 kHz and the impedance was measured at room temperature (RT) with 2 mL of culture medium (pre-warmed at 37 °C).

To establish the cytocompatibility of both the inkjet-printed sensors and the manufacturing setups, a live/dead assay in two independent experimental replicates was used. After 3 days in vitro, the cultures were stained using a solution containing 2 mL of PBS (phosphate buffer saline) with 0.6 µL of calcein-AM (2 µM in PBS) and 2.4 µL ethidium homodimer-1 (4 µM in PBS). The cultures were previously washed with PBS and the staining solution was left to react for 30 min at RT in dark condition. A minimum of 30 images around different sites over the IDEs was taken and analyzed by using both a self-programming script in MatLab and ImageJ software. An average of the proportion of live/dead cells was reported as the cell viability of the assay. To study the cellular adhesion to the sensor SEM was used. Samples were fixed with 2.5% (*v/v*) glutaraldehyde (Sigma Aldrich) in H_2_O distilled at RT for 1h and later washed three times with PBS. Thereafter, samples were dehydrated by passing them through increasingly alcohol dilution for 5 min. Samples were air-dried and gold sputtered before imaging.

### 2.4. Cell Culture

HaCaT cells, a line of immortalized human keratinocytes, were provided from Centro de Investigaciones Energéticas Medioambientales y Tecnólogicas (CIEMAT, Madrid, Spain). Cells were transformed by a green fluorescent protein-expressing retroviral vector to enable fluorescent microscopic observation. Cells were cultured at 37 °C, 38% humidity, 5% CO_2_ in a cell incubator (Shel Lab CO_2_ Serie; Sheldon Mfg. Inc., Sheldon Mfg. Inc., Cornelius, OR, USA) in basal medium (Dulbecco´s Modified Eagle Medium 1.8Mm Ca^2+^, ThermoFisher Scientific) supplemented with 10% (*v/v*) FBS and 2% (*v/v*) penicillin-streptomycin (100,000 U/mL-10,000 μg/mL respectively). The culture medium was changed every 2-3 days. At 80% confluence cells were trypsinized (0.25%-EDTA), followed by centrifugation and plated at a 1:4 dilution.

### 2.5. Preparation of the Inkjet-Printed IDEs-Based Device for Cellular Impedance Monitoring

Before seeding the cells, inkjet-printed IDEs-based setups were washed with a solution of Virkon™ S, cleaned using de-ionized water, and then sterilized by submerging in 70% ethanol (EtOH) for 15 min. EtOH was removed and devices were washed with PBS twice and left in UV exposition for a minimum of 20 min. To promote cell adhesion, the surface of the sensors was functionalized with a solution of 2.5 mL containing collagen (Sigma Aldrich, 0.20%, collagen type II from Calfskin; solution at 1:20 *v/v* in PBS) under UV radiation for 2 h inside a biosecurity cabin (Bio IIA/G, Telstar, Madrid, Spain). The remaining solution was removed, and samples were washed with PBS and air-dried inside the cabins.

### 2.6. Impedance Monitoring Protocol

Impedance was measured with a sinusoidal perturbation of 25 mV in amplitude and no DC bias at 15 points per decade in the frequency range of 100 Hz–1 MHz. Each impedance spectrum was measured with an averaging of three repetitions. As impedance was acquired over multiple hours along with the experiment, a temporal variation in the normalized impedance spectrum was estimated using the dimensionless parameter cell index [36] defined in equation (1), where |Z (0, f_i_)| is the magnitude of the impedance at time 0 (i.e., begging of the experiment) and given frequency *i* along with the number of frequencies N, and |Z (t, f_i_) is the magnitude of the same frequency at a given time point. This adjustment allows observing the relative change in the impedance signal due to the presence of cells. If no cells are in contact with the surfaces of the electrodes, cells are not well-attached, or the number of cells is insufficient to generate a perturbation of the electrical signal, the relative changes in the impedance will be insignificant and therefore, the value of the cell index would remain close to zero:

(1) $${\mathrm{cell}\;\mathrm{index}\;(t)=\max}_{i=1,\ldots ,\;N}\left|\frac{\left|{Z(t,\;f}_{i})\right|-\left|{Z(0,\;f}_{i})\right|}{\left|{Z(0,\;f}_{i})\right|}\right|$$

In addition to the cell index, the cell-substrate impedance was fitted to an electrical circuit model (ZView^®^ software from Scribner Associates, Southern Pines, NC, USA). The fitting quality was evaluated using chi-squared values calculated as the square of the standard deviation between the original data and the calculated spectrum. Fittings performed with x^2^ values lower than 10^−4^ was selected as the indicator of the accuracy of the chosen equivalent circuit. The electrical circuit model and its related physical considerations were obtained by a non-faradic analysis.

### 2.7. Proliferation Assay

For the proliferation assay, different HaCaT-GFP cell densities (12,000 cells/cm^2^; 35,000 cells/cm^2^, 75,000 cells/cm^2^ and 130,000 cell/cm^2^) were seeded on the coated IDEs setups. For monitoring death or detachment 40% and 60% of confluence were seeded. In both sets of experiments, impedance recordings were performed continuously at a time interval of 10–12 h over the entire experimental period. Before seeding the cells, 2 mL of culture medium was added to the IDEs devices and incubated at 37 °C for 20 min to record the background impedance value.

### 2.8. Migration Assay

In the cell migration assay and following the protocol from [37], an artificial wound was created in the cell monolayer using a stencil made of PDMS (3.57 cm^2^). HaCaT-GFP cells were seeded with a density of 1.2∙10^6^ cells (~200,000 cells/cm^2^) that ensured confluence on the non-exposed PDMS area the following day. 24 h after cell seeding the stencil was removed, leaving a free space for cells to migrate. Compared to the scratch assay, this method avoids cell damage at the edge of the scratch caused by the pipette and allows a simplification of the problem analyzing only one half of the wound [38]. The impedance was then measured continuously at a time interval of 6 h over the entire experimental period. Cell images of the boundaries were captured at a time interval of 6 h over the entire experimental period. For each moment, three images were taken at least to calculate the mean value of the velocity of migration.

### 2.9. Image Processing

For the live/dead assay the images of each fluorophore were stored and processed as separate images, and eventually merged into one for a qualitative interpretation. For image analysis and processing either ImageJ (free download from the NIH) or MatLab were used. Cell number was calculated by detecting and counting the cells in the images with the expression of the mean value. For the migration processing, the equation for cell velocity (2) was applied, where υ is the velocity, ${\mathrm{distance}\;}_{t0}$ is the initial distance of the front edge of the cells, and ${\mathrm{distance}\;}_{\mathrm{tf}}$ the distance of the front edge of cells at an observed time:

(2) $$\upsilon =\frac{{\mathrm{Distance}\;}_{\mathrm{tf}}\left(\mu m\right){-\mathrm{Distance}\;}_{t0}\left(\mu m\right)}{\mathrm{Total}\;\mathrm{time}\;\left(h\right)}$$

The recovery degree of the wound was adjusted by the following equation

(3) $$\mathrm{RD}=\frac{{\mathrm{Mean}\;\mathrm{edge}\;\mathrm{displacement}\;}_{\mathrm{tf}}\left(\mu m\right)}{\mathrm{Total}\;\mathrm{displacement}\;\left(\mu m\right)}\times 100$$

where RD is the recovery degree of the wound in percentage, mean edge displacement _tf_ is the displacement of the front cells at an observed time, and total displacement the total area to be recovered by the cells over the sensors.

### 2.10. Triton X-100

To destroy the lipid bilayer in the cell membranes and thus determine the contribution of the cell membranes in the measured impedance at the endpoint of the proliferation assay, a non-ionic detergent (Triton X-100) was used. Triton X-100 (Sigma Aldrich) was dissolved in PBS at 0.1% (*v/v*) final concentration. The culture medium was removed from the samples and 2 mL of the solution containing 0.1% Triton X-100 was added to each sample. The impedance was continuously acquired for 23 min every 60 sec in the range of frequency from 100 Hz–1 MHz. At 23 min, images of the cells on the sensors were taken.

### 2.11. Statistical Methods

For statistical analysis a grouped analysis two-way ANOVA was implemented by Origin v. 9 (OriginLab Corporation, Northampton, MA, USA). P-values higher than 0.05 were not considered as significant differences between samples. A confidence interval of 95% was used. Results from the statistical analysis were depicted in the figures in the form of asterisks.

## 3. Results

The ultimate objective of this work is to assess the feasibility of using inkjet-printed sensors embedded into cell cultures and laboratory-growth skin tissues to monitor the cell´s status using a non-destructive real-time technique. Eventually, sensors and 3D bio-engineered tissues could be printed concomitantly. To that aim and previous to any cellular experiments, the inkjet-printed sensors were characterized to evaluate the quality of the fabrication process (morphological and electrical characterization of the sensors, Section 3.1.1) and to study any toxicity effects associated to both the inkjet-printed sensors and the manufacturing setups (cytocompatibility-based characterization, Section 3.1.2). After this preliminary characterization, the feasibility of using the inkjet-printed sensors to monitor relevant processes of laboratory-growth skin tissues is demonstrated, (Section 3.2) entailing cellular proliferation, detachment, and migration. These sets of cellular experiments also provide evidence of the long-term electrical performances of the sensors embedded inside the cell cultures. The obtained results follow.

### 3.1. Characterization of the Inkjet-Printed Sensors

#### 3.1.1. Morphological and Electrical Characterization of the Inkjet-Printed Sensors

**Figure 3:**
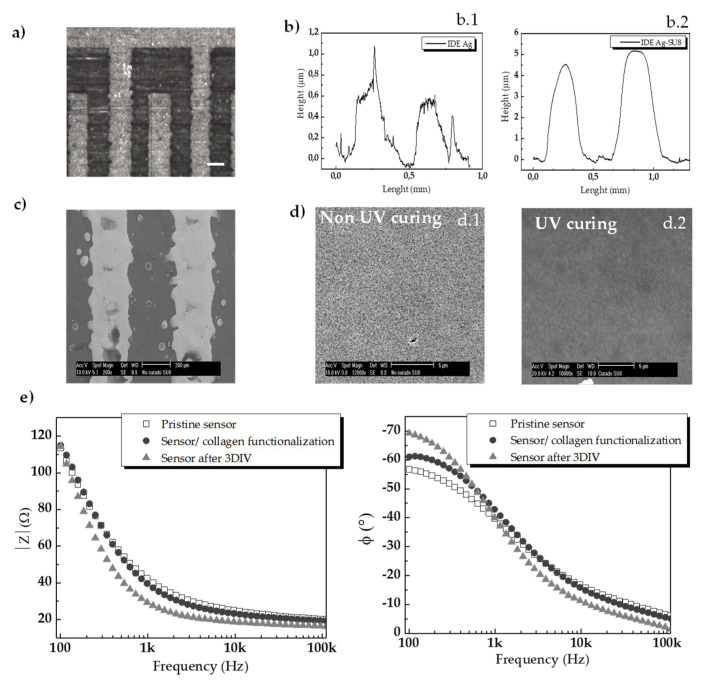
(**a**) Optical micrography of the inkjet-printed sensors (scale bar 100 µm). (**b**) Surface profilometry of the two continuous inkjet-printed electrodes, revealing a thickness of a 0.6 µm for bare Ag electrodes (**b.1**) and 4 µm for passivated electrodes (**b.2**). (**c**) SEM micrography of the inkjet-printed sensor surface in a top view (scale bar 200 µm). (**d**) The effect of UV curing on the SU-8 passivation layers showed an increase in the smoothness of the outmost layer. UV treated samples showed a smoother surface (**d.2**) compared with non-treated samples (**d.1**). (**e**) Electrical performance of pristine inkjet-printed sensors, sensors with collagen functionalization, and sensors after three days in vitro with HaCaT cell cultures. Magnitude of the impedance (left) and phase (right).

The planar interdigitated electrodes (IDE) and passive layers printed on flexible polymer substrates (PET) were morphologically characterized by optical microscopy, SEM, a profilometer. The width of the fingers of the interdigitated sensors was measured by image processing that resulted in an average electrode size of 300 µm ± 0.1 µm (*n* = 66) and satellite drops of an average diameter of 20 µm ± 15 µm (Figure 3a,c).

A drop spacing (DS) of 15 µm selected to print the conductive ink resulted in a thickness of approximately 600 nm (Figure 3(b.1)). The DS of 20 µm adjusted to print the SU-8 ink generated a thickness of about 1 µm for one single printed layer. As the passivation layer was printed in a total of three-layer, the total thickness resulted in about 3 µm (Figure 3(b.2)). A single SU-8 layer is about 300 nm thicker than the electrode layer, which may be explained with the differences in the amount of solvent and compositions in each ink formulation. In bioelectronics, 3D nano-topographies compared to planar topographies have shown to improve cell adhesions, explained by the contact guidance phenomenon [39,40]. Tight adherences between the tissue and/or cells and electronics are highly desirable to record signals from the adhered cells with a high signal-to-noise ratio. Thus, the topographic feature obtained in our sensors may promote the adhesion of cell cultures since it simulated the appearance of microgrooves and ridges with dimensions of 300 µm and a total depth of approximately 4 µm.

One of the main concerns in the use of flexible substrate for electronic sensors follows from delamination issues. Potential delamination of the conductive printed lines from the PET substrate as well as the upper passivation and the silver electrodes were characterized by means of SEM (Figure 3c). Figure 3c shows a top view of an SEM image of the inkjet-printed structure onto the PET substrate. Inkjet-printed silver lines and substrate smoothly attached without any visual delamination. The conductive electrodes appeared as a second layer on top of the substrate and the non-conductive layers cannot be distinguished. On the other hand, since the outmost layer of the passivation is the surface directly in contact with the cells, the effect of UV curing the SU-8 ink before cell seeding was studied by SEM. Surfaces of the SU8 ink cured with UV are smother compared with non-cured surfaces (Figure 3d).

This effect could be explained by the fact that UV light polymerizes SU-8 ink formulation by cross-linking. Considering that smooth surfaces may hinder cell adhesions and the surfaces of the sensors will be functionalized next with extracellular components (collagen) (see Section 2. Materials and Methods, Section 2.5), we have selected the condition that renders surfaces of the sensors less smother and more reliable for promoting cellular adhesion. In other words, for the cellular experiments we did not treat sensors with this later UV curing (see the next Section 3.1.2 for cytocompatibility).

Electrochemical impedance spectroscopy (EIS) was used to evaluate the electrical properties of the devices. Impedance curves presented a capacitive-like behavior for lower frequencies until 5 kHz (phase angle = −70° at 100 Hz) which flattened at higher frequencies, corresponding to a resistive-like response (phase angle near to 0°). The low frequency (capacitive) behavior, which covers most of the frequency spectrum, is due to the double-layer capacitance at the electrode interface caused by the absorption of ions/molecules from the culture medium into the surface of the sensors. The high frequency (resistive) behavior is representative of the solution resistance. The impedance observed at 100 Hz showed an average value of 120 Ω (*n* = 3, σ = 10 Ω) for pristine devices (i.e., before both cell seeding and collagen functionalization). After collagen functionalization, the average impedance magnitude did not significantly change, but the phase angle varied an average of ∆5° at 100 Hz. The long-term electrical stability was tested by measuring EIS after performing cellular experiments. After 3 days in vitro (3 DIV) with cell cultures, the impedance exhibited a variation in the magnitude values and phase for the frequencies above 500 Hz–1 kHz. The average deviation in impedance magnitude at 1 kHz was about 10 Ω (σ = 0.65 Ω, *n* = 3), while phase varied ~15° (σ = 7°, *n* = 3). Regardless of these slight variations, the electrical characterization that is shown in Figure 3e demonstrated a good electrical performance of the sensor using inkjet-printing electrodes for in vitro monitoring of cell cultures, as it will be shown in Section 3.2.

#### 3.1.2. Cytocompatibility Evaluation of the Inkjet-Printed-Based Device

**Figure 4:**
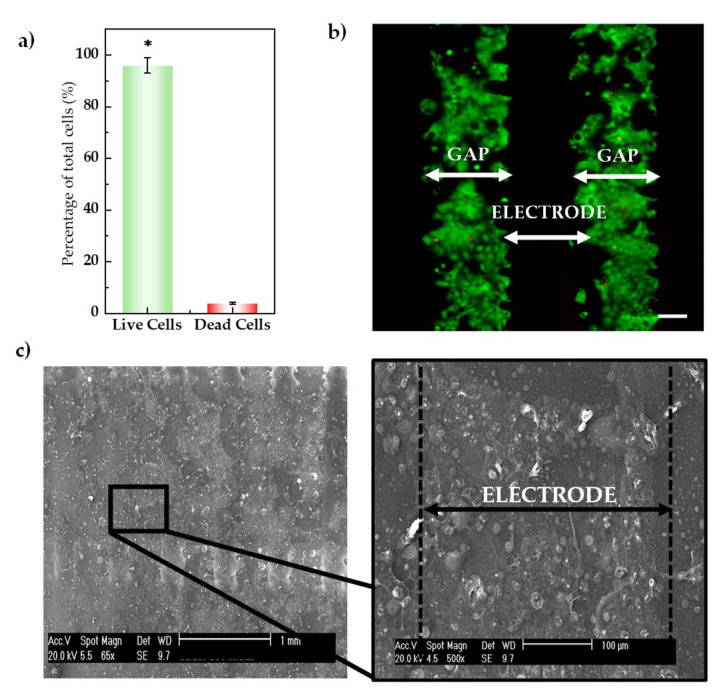
(**a**) Live/dead assay on the HaCaT cell line after 3 days of incubation. Distribution of live and dead cells of a total of 60 images of areas around different electrode sites on IDEs was analyzed. The error bars represent the observed standard deviations. (**b**) Fluorescent image of the surface of two consecutive fingers of the electrodes (scale bar 100 μm) showing a simultaneous detection of the live and dead cells is observed, in which the live cells can be seen in green while the red spots identify dead cells. Owing to the non-transparency of the conductive lines (AgNP), stained cells can be observed only on the gaps of the interdigitated electrodes, as the sample was illuminated from below. (**c**) SEM image of the IDEs covered by a confluent layer of HaCaT cells (scale bar 1 mm), confirming that when stained cells are observed in the gaps of the interdigitated fingers, they were also over the electrode lines. The magnification of one area is highlighted showing cells adhered to the surface of the electrode (scale bar 100 μm).

Results from the study of potential toxicity effects of the inkjet-printed devices on the cell cultures after 3 DIV (assessed in a total of two experimental repetitions are depicted in Figure 4a. The graph represents the total percentage of live and dead cells and corresponds to the mean and standard deviations of the samples of each repetition. As it is shown, the number of living cells was significantly superior to the dead cells (an average of 96% for the former and 3% for the latter; *n* = 60, *p* < 0.005).

An example of stained cells seeded on the two consecutive fingers of the sensor is depicted in Figure 4b, in which green fluorescence represents live cells while red fluorescence represents dead cells. Due to the non-transparency of the conductive lines and that the samples were illuminated from below, stained cells can be observed only on the gaps between the interdigitated electrodes. To confirm that cell are also attached over the area of the electrodes (dark zone in the staining images—Figure 4b—where the fluorescence studies cannot be performed due to the opacity of the sensors), the experimental conditions of the live/dead assay were replicated to observe the cells over the IDE surface by SEM. A top view of an electrode section covered with a confluent cell monolayer of HaCaT cells is depicted in Figure 4c. Cells appeared homogeneously distributed over the sensor surface and keep their round normal morphology. In the expanded micrography (Figure 4c, right) is observed both cell-cell and cell-substrate tight adherence. Thereby, it can be confirmed that when cells were observed in the gaps of the IDEs fingers by fluorescence staining, they were veiled over the opaque electrodes. Such a tight interface between the sensor and cell culture reduces the parasitic contribution that may come from the lack of stability of the adherent cells on the surface of the sensors, which is essential to ensure a high quality of the impedance signal recording.

Given the results previously described in the live/dead staining cells in which quantification of live and dead cells indicated a statistical significance living cells and the confirmation of cells over the surface of the sensors by SEM, it can be stated that the inkjet-printed IDEs and the custom packaged devices are cytocompatible.

### 3.2. Impedance Monitoring of 2D Epithelial Cultures

Results from the integration of the inkjet-printed sensors into cellular cultures for monitoring proliferation, detachment, and migration of a monolayer of a cell line of keratinocytes using impedance spectroscopy are presented in this section. We have measured the impedance of the electrodes embedded in the cell cultures in a frequency range of 100 Hz–1 MHz, which comprises the region of β biological dispersion [9,41]. At these frequencies, the extracellular resistance and membrane capacitance are the primary contributions to the impedance changes both in magnitude and phase [42].

#### 3.2.1. Demonstration of the Use of Inkjet-Printed Sensors for Impedance-Based Monitoring of Cell Proliferation

**Figure 5:**
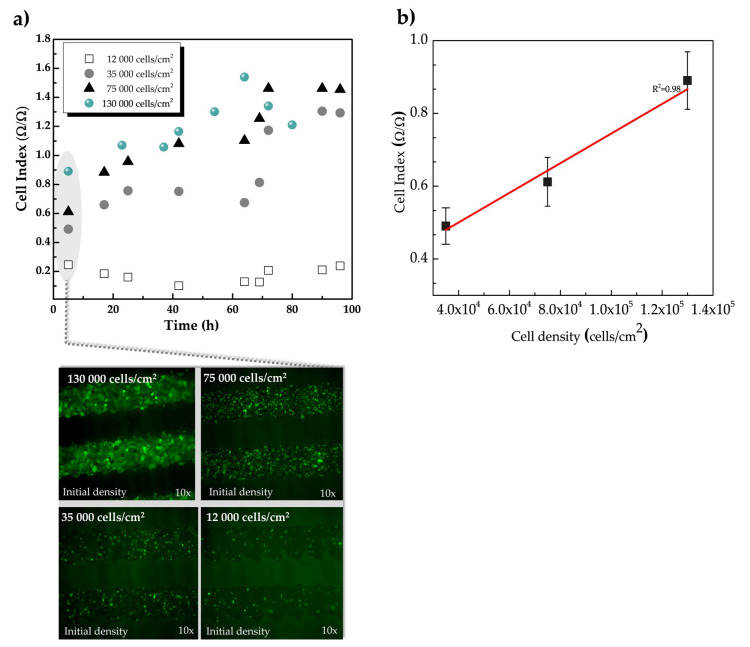
Monitoring of proliferation and determining detection limits of the sensor at 100 Hz. (**a**) Cell index values versus time in hours for real-time impedance monitoring of HaCaT-GFP cell adhesion and proliferation over 96 h. Cells were seeded at different initial densities (12,000 cells/cm^2^; 35,000 cells/cm^2^, 75,000 cells/cm^2^ and 130,000 cells/cm^2^) on collagen-coated inkjet-printed IDEs. The higher the initial cell densities, the higher the cell index obtained. For the initial cell densities of 75,000 cells/cm^2^, the maximum cell index value is reached at around 72 h, indicating cell confluence in the culture. For low initial cell density (12,000 cells/cm^2^), the measurements are significantly lower than the other cultures, remaining low even after 69 h, and slowly increasing after that. For the highest initial cell density (130,000 cells/cm^2^), the maximum cell-index value is detected around 60 h, sharply decreasing in the following measurements. As cultures with different cell densities initially seeded have different growth rates, the maximum cell index is expected to be detected at different time points, which is verified in the cell index curve. (**b**) Cell index linearly correlated with the number of cells initially seeded on the sensors between 35,000 and 130,000 cells/cm^2^ at 17 h after seeding. (error bar represents the standard deviation, *n* = 3). As cell index of the cultures with the lowest cell densities (i.e., 12,000 cells/cm^2^) remained low along with 69 h, such values were not included to analyze the correlation between initial cell densities and the cell index, as we assumed that was below the sensitivity limit of the device.

Results obtained from the changes in the impedance expressed using the dimensionless parameter cell index (Materials and Method Section 2.6) confirmed a temporal variation in the impedance´s magnitude due to the presence of cells. We have investigated the correlation between the cell density and the cell index values of the impedance by seeding different HaCaT-GFP initial cell densities (12,000, 35,000, 75,000 and 130,000 cells/cm^2^). The impedance of the samples with different cell densities was continuously monitored for up to 96 h. Figure 5a shows the cell index values of HaCaT-GFP cells caused by the adhesion and cell proliferation during 96 h of impedance monitoring. The representative cell index curve shown was obtained at 100 Hz, however, the same tendency is observed for the whole frequency measurement range. In other words, we did not observe a specific sensitive frequency to detect the changes in the impedance magnitude caused by the cells. The evolution of the different initial cell densities is shown below in Figure 5a. A micrographic of the cultures with different initial cell density is shown at the bottom plot of Figure 5a.

As seen in Figure 5a, cell adhesion and spreading induced a rapid cell index increment and a significant difference between the four densities initially seeded. This means that the higher the number of cells both initially seeded and in contact with the sensor, the larger the variation of the electrical impedance detected before and after seeding the cells. Therefore, the initial value of the cell index (relative changes in the impedance measured at different time points) varied depending on the cell density. In the case of samples with higher initial cell density (130,000 cell/cm^2^, 75,000 cells/cm^2^ and 35,000 cell/cm^2^), the increment was detected within the first hours and was determined as an indication of the proliferation along the experimental period. Such a relation between impedance responses and cell densities was established with the assistance of microscopy analysis, as is depicted in the lower part of Figure 5a. For the highest cell density initially seeded (130,000 cells/cm^2^), the cell index reached is maximum value around 60 h from cell seeded (1.54 cell index units). For the initial cell density of 75,000 cell/cm^2^, the cell index reached its maximum value around 72 h (1.5 cell index units) that kept constant during the last 24 h. This difference in the maximum cell index at different time points can be explained with the fact that cultures with different cell densities initially seeded have different dynamics in their growth rates. In other words, the larger the cell population seeded at one point in time, the larger the cell population after a certain hour in time. When the number of cells in a determined area, however, reaches a density that led cells to contact each other, the proliferation processes may be altered and the growth rate is not homogenously constant anymore, which is explained by a phenomenon known as contact inhibition.

This behavior is observed where the relative increment in the cell index at the different measured time points is lower in the samples with 130,000 cells/cm^2^ initially seeded than in the samples with 75,000 cells/cm^2^ and 35,000 cells/cm^2^. Cell index for the lowest cell density is significantly lower than the other higher densities cultures, remaining low even after 69 h and slowly increasing after that. Cells seeded on the electrodes surface yield an increment in the interface impedance mostly owning to the high insulating properties of the cell membrane. Whether the number of cells is not sufficient to generate a perturbation in the electric field on the surface of the sensor no changes can be observed in the impedance due to the presence of cells, and therefore in the cell index. Under such conditions, measurements are below the detection limit, and the resistance of the solution prevails over the effect of cells in the impedance, fixing a lower limit in cell density for the impedance measurement.

To characterize this influence on the measured impedance associated with cell proliferation, we have defined the sensitivity of the sensors as the ratio S = ∆cell index/∆cell density as the cell index value reflected a linear correlation with the number of the initially seeded cells on the electrodes (R^2^ = 0.92). The obtained sensitivity (Figure 5b) was S = 4.36 cell index/cells∙cm^−2^ which was extracted from the slope of the lineal fit. The curve shown in Figure 5b was obtained at time 17 h of the experiment, but the same tendency can be extracted from the different points of curve in Figure 5a. Thus, it can be considered that the cell index value mirrors the total number of adhered cells initially seeded, in agreement with what has been previously reported [43,44].

##### Study of the Contribution of the Cell Membranes Electrical Characteristics in the Impedance at the Endpoint of the Proliferation Assay

**Figure 6:**
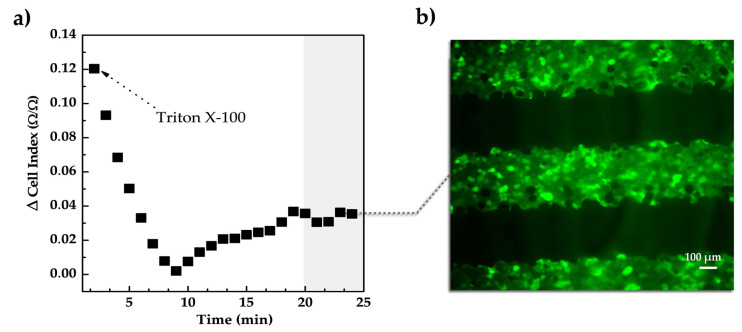
(**a**) Monitoring the addition of chemicals by impedance spectroscopy. Triton X-100 was added to the cell culture to destroy the bi-lipid membrane of the cells. The impedance drastically dropped in the first 10 min after the addition of the chemical. (**b**) Optically observation of the presence of cells under the sensors after 20 min of adding Triton X100.

Results obtained by analyzing the contribution of the electrical properties of the cell membrane (i.e., isolating property under an electric field) on the measured impedance demonstrated that the cell membranes disruption correlates with the impedance changes at the endpoint of the proliferation assay. We have performed this study since impedimetric-based cell measurements can be influenced by variations in the medium composition as a product of cellular metabolism and from the alterations in the impedance at the transducer surface as an effect of the isolated cell membrane in direct contact with the sensor. The loss of the isolating properties of the cells that are in contact with the surface of the sensors should result in a variation on the sensor signal. Triton X-100 is a non-ionic detergent widely used in cellular assays to physically disrupt the lipid bilayer of the cell membranes. The addition of Triton X-100 was used at the end of the proliferation experiment to determine the influence of the cell membrane electrical characteristics in the measured impedance. Figure 6a shows the values of the cell index for 25 min after adding Triton X-100 (10% *v/v* in PBS) in cell cultures. Cell index drastically drops down within the first 10 min after the addition of such chemical, varying a total of 0.12 units. During the last 10 min of monitoring, the cell index varied by 0.04 units. 10 min is the amount of time usually used when performing cellular studies using Triton X-100. 

Cells were optically observed after 20 min of adding Triton X100 to assess that the addition of the chemical did not detach cells from the sensor surface (Figure 6b). In this sense, the variation in the cell index and therefore, in the impedance measured was attributed to the destruction of the cell membranes resulting in a complete loss of their isolating function [45]. Intact cell membranes (before adding Triton X-100) produced a greater obstacle for the electric field compared with non-intact cells, which generated the differences in the impedance. This way we can evaluate what is the actual contribution to the total measured impedance from the two factors mentioned above.

#### 3.2.2. Demonstration of the Use of Inkjet-Printed Sensors for Impedance-Based Monitoring of Cell Detachment or Death

To emulate the cellular cycle of shedding skin once a month, we have performed a cell detachment (or death) study by seeding high cell densities (60% of confluence) on the IDEs devices and monitoring the impedance over 80 h continuously. In this study, we have used the measured impedance for the whole frequency range, this is, from 100 Hz–1 MHz, instead of a single frequency point as used before. For this reason, we have used the Nyquist plot to display the measured impedance for an easier analysis since each point of the plot represents the impedance (real part and imaginary part) at one frequency. Besides this, and for in-depth analysis of the 2D tissue-electrode interface, magnitude and phase angle spectra were fitted to an electrical equivalent circuit.

**Figure 7:**
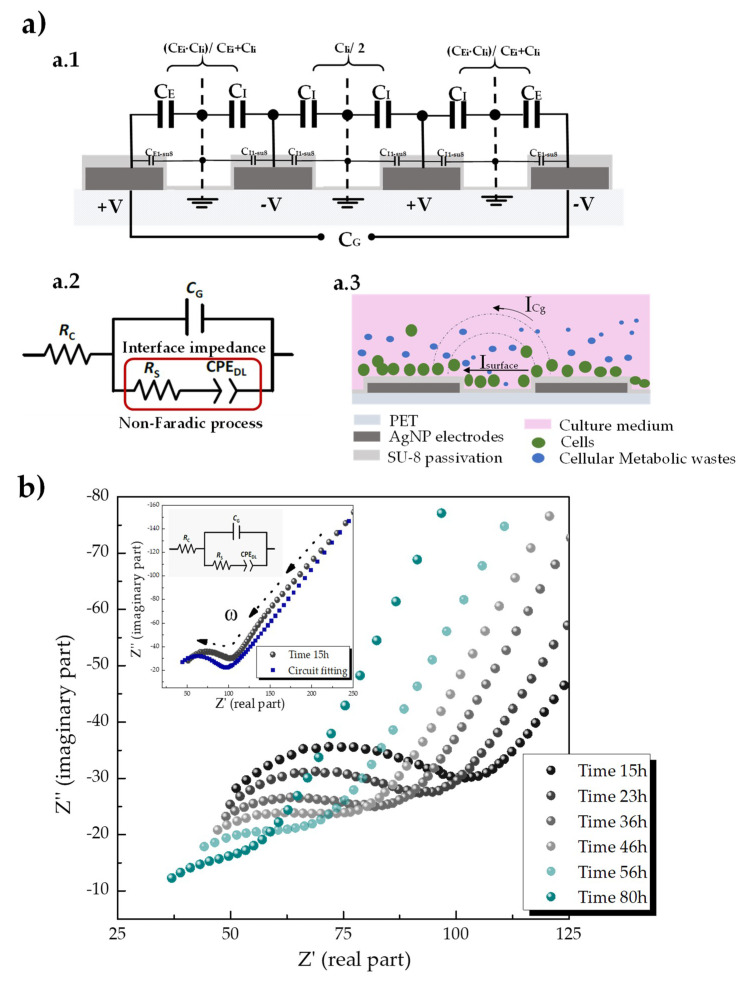
(**a**) Equivalent circuit model to fit the impedance measured in a non-faradic analysis. (**a.1**) Example of the electrical equivalent applying partial capacitance techniques of a sensor with four electrodes and considering the effect of finite multilayer passivation on top of the electrodes on the static capacitance. (**a.2**) Electric circuit model of the IDEs with cells in a culture medium modeling the resistivity of the culture medium, Rc, the capacitance of the IDEs, Cg, and the impedance of the surface interface. The presence of cells is modeled by a constant phase element, CPEd_DL_, and a resistance Rs. (**a.3**) Schematic representation of the biophysical interpretation of such an equivalent circuit model showed in a.1. The total current can pass through the interface as a sum of the contributions from the geometrical capacitance of the IDEs (curved arrow I_CG_) and the non-faradic process that takes place in the interface of the electrodes due to the presence of cells (straight arrow I_surface_). The dashes lines in the schematic represent the electric field generated due to the nature of the interdigitated electrodes. Green circles represent the HaCaT-GFP cells seeded on the sensors while the blue dots represent the cellular wastes as a result of their growth medium’s consumption. (**b**) Nyquist plot of experimental impedance spectra for monitoring cell detachment and death during 80h period. The inset plot shows the measured impedance and the corresponding fitting with the circuit model at time 15 h. Each point in the Nyquist plot represents the impedance at one frequency, in which higher frequencies appear closer to the origin (i.e., lower values of the real part in the magnitude of the impedance).

The impedance spectra alterations of the IDEs devices can be modeled either under Faradic or non-Faradic processes [46]. We have analyzed the interaction in the non-Faradic process since current flows as a result of the capacitive nature of the electrodes and changes in the impedance are based on changes in the capacitance between the interdigitated electrodes. In addition, conformal mapping and partial capacitance technique can be used to include the effect of a finite, multi-layer, passivation over the electrodes in the capacitance of the devices [47,48], which result in an equivalent circuit as shown in Figure 7(a.1). From the structure drawn in Figure 7(a.1), the analytical expression for the total capacitance Cg can be obtained as the parallel of the contribution from the grouped capacitances in each layer (air, SU8) that result from considering the ground electric walls in the halfway between the electrodes (V = 0) [47]. For the sake of simplicity, the capacitance of this multilayer structure is referred to as Cg. Under these assumptions, cells-sensor interaction may be modeled by the electrical equivalent circuit presented in Figure 7(a.2).

To explain and therefore understand the equivalent circuit that electrically behaves like the empirical impedance data (Figure 7(a.2)), we have based the interpretations upon the angle of view of the schematic drawing in Figure 7(a.3). According to this schematic drawing, the total current can pass through the interface as a sum of the contributions from the geometrical capacitance of the IDEs (curved arrow I_Cg_) and the non-faradic process that take place in the interface of the electrodes due to the presence of cells (straight arrow I_surface_). On the other hand, equivalent electrical circuits not only must fit the experimental data, but they also should represent the system in question in the first order. Modeling cell-sensor interactions encounter challenges due to the own nature of the biological complex system. Cell membranes (ideally modeled as capacitors) comprise a lipid bilayer and integrated ion channels that can open and close to exchange cellular signals, cell-substrate adherences are dependent on focal adhesions (complex structures conformed by proteins), and cell growth medium contains animal serum. All these elements with different electrical properties neither will be completely reproducible nor by using an exclusively real electrical circuit [12,49]. Under these considerations, we have allowed the electrical component to describe cells to be non-ideal (i.e., frequency-dependent), thereby modeled by a constant phase element (CPE).

To model the capacitance between the interdigitated electrodes considering the passivation layer on top (Figure 7(a.1)), the stray capacitor C_g_ was used. A process that may occur physically in parallel were modeled by using a parallel model. For example, we have assumed that the total current through the working interface was the sum of distinct independent contributions from the non-faradic process and the stray capacitor. Finally, electrolytes were modeled by resistances. Hence the solution resistance R_c_ represents the culture medium, which was inserted as a series element since all the current must pass through it. The presence of cells prompts alterations in the ionic environment around the electrode at the impedance interface which was modeled by R_s_ that account for the resistance of the medium between two electrodes.

A Nyquist plot of the experimental impedance spectra obtained for the monitoring of cell detachment and death is shown in Figure 7b. Using the equivalent circuit model represented in Figure 7(a.1) and described above to fit the obtained impedance spectra allowed to make the following analysis. First, we can clearly see that the Nyquist diagram (Figure 7b) consists of two main domains: a semicircle at high frequencies (Z′ < 100; Z″ > −40) and a straight line (at around 45° angle) at low frequencies (Z′ > 100; Z″ < −40). The semicircular part of the Nyquist diagram is where the parallel between the resistance R_s_ and the stray capacitance C_g_ account for the biggest part of the impedance. The intercept of this semicircle with the Z′-axes gives (on the left side) the value for R_c_, while the intersection on the right side gives the values for R_s_. In the model, the solution resistivity R_c_ is expected to be constant (or barely vary) because the culture media solution was carefully prepared identically for all experiments and the same amount was added to every sample each time. In this sense, when cells reach confluence, contact inhibition may lead cells to detach to the surface of the sensor, and thus, cell-substrate adherences losses their tight junctions (become weaker) leading to lower R_s_. Second, as we can see in Figure 7b, as time increases a lower surface resistance gives rise due to loss of surface charges (i.e., fewer cells) at the surface of the sensor. In other words, at time 15h the adherent negatively charged of HaCaT-GFP cell yields a surface impedance significantly greater compared with that one at time 80 h. By fitting the impedance measurements to the equivalent electric circuit model, ∆R_s_ (Ω) in the interface varied from 57 Ω at 15 h to 24 Ω at 80 h, while ∆CPE(F) from 150 µF at 15 h to 0.76 µF at 80 h. This experiment demonstrates the use of the inkjet-printed sensors and the impedance-based analysis for monitoring of cellular detachment (or death) processes.

#### 3.2.3. Preliminary Use of Inkjet-Printed Sensors for Impedance-Based Monitoring of Cell Migration

Finally, to study a third relevant cellular process for skin-based sensing applications, a preliminary study to validate the developed sensors for monitoring of cell migration was performed. In this experiment, an artificial wound was created in the cell monolayer using a stencil made of PDMS and cell migration was tracked for at least 80 h using both impedance spectroscopy and fluorescence microscopy at once. 

**Figure 8:**
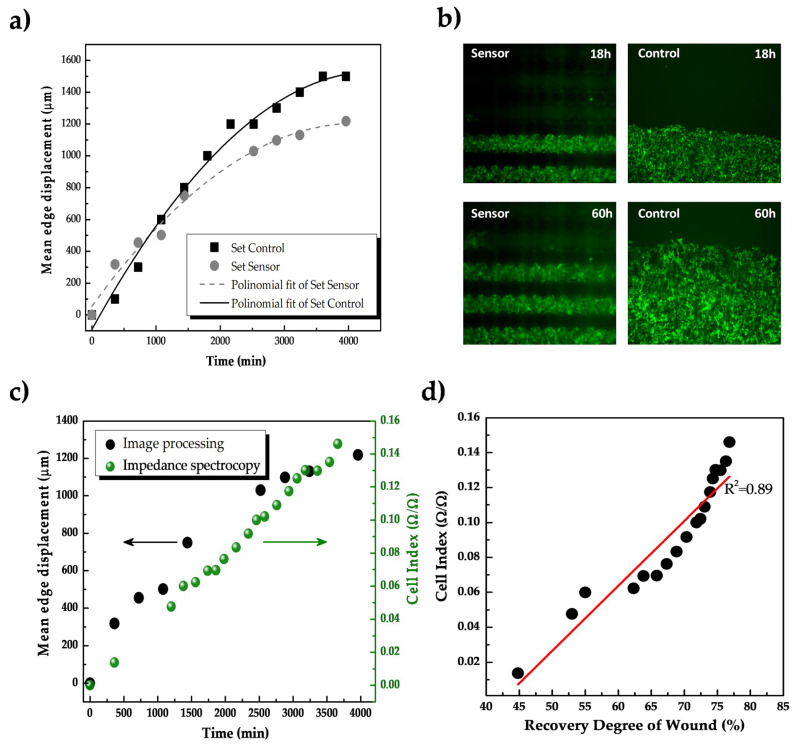
Monitoring of cell migration using real-time impedance spectroscopy. (**a**) Mean edge displacement of a monolayer of cell cultures in control groups (Petri dish) and experimental groups (cells seeded on the IDEs devices) during 4000 min of time-lapse observation. (**b**) Visualization of the experiment under a microscope. We can see how both for the sensors and the control cultures the cell edge advance towards the top of the figure in time (migration). Note how in the sensors culture the electrodes appear as dark stripes due to the opacity of the inks (illumination from below) (**c**) The relation between real-time impedance monitoring of cell migration and the displacement of cells by image processing of the microscope images. (**d**) Linear correlation between the cell index values and the recovery degree of wound healing, with a linear regression coefficient R = 0.89.

Mean edge displacement in the monolayer of cells in control groups and samples seeded on the sensors is depicted in Figure 8a, obtained by leading-edge detection using image processing (Figure 8b). 

The mean edge displacement obtained for control groups was around 1400 µm at 60 h, while the displacement of cells seeded on the IDEs sensors reached 1200 µm at 60 h (see Figure 8b for a visual illustration of the migration experiment). Different velocity in the cell migration was obtained in controls (0.35 µm/min) compared with the experimental group (0.3 µm/min), which may be explained due to the differences in the topography in the controls (Petri dish) and the sensor’s surface (see Section 3.1). Moreover, the image processing for both sets of groups was slightly different due to the discontinuities of the fluorescence images when cells migrated over the conductive lines in the sensor (dark zones in Figure 8b). Impedance spectra were continuously acquired every three hours during 66 h and further processed using the cell index parameter.

The relationship between the cell index value and the mean edge displacement in the sensor group is depicted in Figure 8c. Cell index value varied a total of 0.15 units during the experiment, showing an increment of about 0.00125 (Ω/Ω)/µm. To facilitate the interpretation of the impedance response of the sensor during the recovery of the wound, the mean edge displacement of the cell monolayer was adjusted to a recovery degree of the total simulated wound. The recovery degree was calculated by a relation with the total area to be recovered by cells and the mean edge displacement at a specific moment in time. Figure 8d shows the continuous increment in the cell index during wound healing, where a linear correlation (R^2^ = 0.889) between the cell migration and impedance changes was established. Consequently, the impedance signals of the sensors in the response of the HaCaT cell migration have reflected the healing degree without the need for visual observation.

## 4. Discussion and Conclusions

In this work, we have demonstrated the feasibility of the integration of inkjet-printed IDEs into 2D epithelial cultures for in real-time, label-free monitoring proliferation, detachment and migration of keratinocytes by impedance spectroscopy. The sensors consisted of interdigitated electrodes with silver nanoparticles as the only conductive element and SU-8 as the passivation layer, which were patterned on a flexible polymer substrate in ambient conditions. The fabrication method is compatible with large-scale production and gives the possibility of engineering a platform that can print sensors/actuators and living cells interactively. Overcoming the bottleneck of the stick rheological/physiological conditions that bio-inks need to meet and fruitful collaborations between materials scientists, biologic and engineers could unleash progress in this envisioned technology.

Our impedance sensors are inexpensive, of rapid prototyping and more mechanically compliant with human tissues than the flat, conventional wafer-based impedance devices. The use of flexible substrates enables cell-substrate impedance chips to overcome the significant mechanical mismatch between the traditionally typical materials used (i.e., glass/silicon) and cells [50]. Regarding impedance-based devices for monitoring cell cultures, only a meager fraction of those sensors have been fabricated by inkjet-printing technology [26] compared to a body of literature that uses the flat and rigid conventional wafer-based method.

Form the morphological characterization, the micro-topographical features of the IDEs resulted in a non-planar surface simulating micro-grove of 300 µm width and height of around 5 µm, which was found to promote tight electronic-tissue interfaces, a prerequisite to either electrical stimulate or record signals with a high signal-to-noise ratio. To improve printing resolutions, some properties during the printing processes [51], technical modifications in the printer setup [24], or tuning the wettability of the substrate [52] can be further considered and optimized. The typical sheet resistance for inkjet-printed AgNP reported in the literature is around 0.4 Ω/□ [53]. According to the total area of the printed structure, the total resistance should be theoretically of 88 Ω. From the electrical characterization, the impedance of the sensors resulted in the same order of magnitude of the theoretical resistance (113 Ω for the pristine device at 100 Hz). The impedance-based analysis of the sensors after 3 days in vitro showed a small variation of ∆10 Ω at 1 kHz, which demonstrated the possibility to reuse the inkjet-printed sensors. Coating the devices with an extracellular component (i.e., collagen) showed that such extracellular protein did not influence the performance of the IDEs inkjet-sensors.

The inkjet-printed sensors and the custom sensors-based unit to culture cells for cell-substrate impedance recording were cytocompatible. The sensor sensitivity to culture’s cellular density was obtained by seeding different cell densities and the relationship between impedance data and cell status was supported with fluorescence microscopy observation. Results showed a sensitivity of 4.36 cell-index units/cells∙cm^−2^ with a linear regression of R^2^ = 0.98 between the impedance variations and the initial cell density. This linearity suggested that the detected impedance mirrored the cell number to an extent and demonstrated the possibility to discriminate against different cell concentrations. To increase the sensitivity of the sensor design, thickness and materials of the electrodes can be optimized in the future [51,54,55,56]. Designing the IDEs spacing on the order of cell size [57] and a thicker passivation layer [58] can also be adopted to increase the sensitivity to the presence of cells.

Finally, experiments of cellular proliferation and migration demonstrated the feasibility of using printed IDEs and impedance spectroscopy for monitoring these two processes, evidencing the long-term electrical performance of the sensors embedded in cellular conditions. The electrical signal of the sensors lineally correlated to the progression of wound healing (linear regression coefficient R^2^ = 0.889). On the other hand, changes in the cell membranes permeability by adding a detergent [9,45,59] correlated with a decrease in the impedance, which demonstrated the rapid response of impedance-based methods to changes in cell structures or tissues.

In conclusion, we have presented bioimpedance inkjet-printed sensors as an inexpensive, label-free solution for real-time, label-free monitoring of anchorage-dependent cells. A non-invasive, real-time quantitative feedbacks of living skin tissues not only represents a promising approach for monitoring wound healing and laboratory-growth skin substitutes but mostly important demands a fabrication method for rapid and easy sensors prototyping. Our sensors, with a sensitivity of 4.36 cell-index units/cells∙cm^−2^, are easily integrable in microfluidics and/or lab-on-chip devices and support the low-cost inkjet printing fabrication as an emerging technology for the resource-limited environment yet massive production.

## References

[1] Cubo N., Garcia M., del Cañizo J.F., Velasco D., Jorcano J.L. (2017). 3D bioprinting of functional human skin: Production and in vivo analysis. Biofabrication.

[2] Wang B., Baeuscher M., Hu X., Woehrmann M., Becker K., Juergensen N., Hubl M., Mackowiak P., Schneider-Ramelow M., Lang K.D. (2020). Development and Characterization of a Novel Low-Cost Water-Level and Water Quality Monitoring Sensor by Using Enhanced Screen Printing Technology with PEDOT: PSS. Micromachines.

[3] Mojena-Medina D., Martínez-Hernández M., de la Fuente M., García-Isla G., Posada J., Jorcano J.L., Acedo P. (2020). Design, Implementation, and Validation of a Piezoelectric Device to Study the Effects of Dynamic Mechanical Stimulation on Cell Proliferation, Migration and Morphology. Sensors.

[4] Feiner R., Dvir T. (2020). Engineering smart hybrid tissues with built-in electronics. Iscience.

[5] Gómez C., Galán J.M., Torrero V., Ferreiro I., Pérez D., Palao R., Martínez E., Llames S., Meana A., Holguín P. (2011). Use of an autologous bioengineered composite skin in extensive burns: Clinical and functional outcomes. A multicentric study. Burns.

[6] Mohammed A., Binder K.W., Murphy S.V., Kim J., Qasem S.A., Zhao W., Tan J., El-Amin I.B., Dice D.D., Marco J. (2019). In Situ Bioprinting of Autologous Skin Cells Accelerates Wound Healing of Extensive Excisional Full-Thickness Wounds. Sci. Rep..

[7] Dargaville T.R., Farrugia B.L., Broadbent J.A., Pace S., Upton Z., Voelcker N.H. (2013). Sensors and imaging for wound healing: A review. Biosens. Bioelectron..

[8] Grimenes S., Martinsen Ø.G. (2015). Bioimpedance and Bioelectricty Basics.

[9] Xu Y., Xie X., Duan Y., Wang L., Cheng Z., Cheng J. (2016). A review of impedance measurements of whole cells. Biosens. Bioelectron..

[10] Grossi M., Riccò B. (2017). Electrical impedance spectroscopy for biological analysis and food characterization: A review. J. Sens. Sens. Syst..

[11] Lo C.M., Keese C.R., Giaever I. (1995). Impedance analysis of MDCK cells measured by electric cell-substrate impedance sensing. Biophys. J..

[12] Ehret R., Baumann W., Brischwein M., Schwinde A., Stegbauer K., Wolf B. (1997). Monitoring of cellular behaviour by impedance measurements on interdigitated electrode structures. Biosens. Bioelectron..

[13] Ehret R., Baumann W., Brischwein M., Schwinde A., Wolf B. (1998). On-line control of cellular adhesion with impedance measurements using interdigitated electrode structures. Med. Biol. Eng. Comput..

[14] Discher D.E., Janmey P., Wang Y.-L. (2005). Tissue cells feel and respond to the stiffness of their substrate. Science.

[15] Tonello S., Bianchetti A., Braga S., Almici C., Marini M., Piovani G., Guindani M., Dey K., Sartore L., Re F. (2020). Impedance-Based Monitoring of Mesenchymal Stromal Cell Three-Dimensional Proliferation Using Aerosol Jet Printed Sensors: A Tissue Engineering Application. Materials.

[16] Marziano M., Tonello S., Cantù E., Abate G., Vezzoli M., Rungratanawanich W., Serpelloni M., Lopomo N.F., Memo M., Sardini E. (2019). Monitoring Caco-2 to enterocyte-like cells differentiation by means of electric impedance analysis on printed sensors. Biochim. Biophys. Acta Gen. Subj..

[17] Kim Y., Kim J.W., Kim J., Noh M. (2017). A novel fabrication method of Parylene-based microelectrodes utilizing inkjet printing. Sens. Actuators B Chem..

[18] Blaschke B.M., Lottner M., Drieschner S., Calia A.B., Stoiber K., Rousseau L., Lissourges G., Garrido J.A. (2016). Flexible graphene transistors for recording cell action potentials. 2D Mater..

[19] Brischwein M., Herrmann S., Vonau W., Berthold F., Grothe H., Motrescu E.R., Wolf B. (2006). Electric cell-substrate impedance sensing with screen printed electrode structures. Lab Chip.

[20] Delaney J.T., Smith P.J., Schubert U.S. (2009). Inkjet printing of proteins. Soft Matter.

[21] Derby B. (2008). Bioprinting: Inkjet printing proteins and hybrid cell-containing materials and structures. J. Mater. Chem..

[22] Esfandyarpour R., Didonato M.J., Yang Y., Durmus N.G., Harris J.S., Davis R.W. (2017). Multifunctional, inexpensive, and reusable nanoparticle-printed biochip for cell manipulation and diagnosis. Proc. Natl. Acad. Sci. USA.

[23] Garma L.D., Ferrari L.M., Scognamiglio P., Greco F., Santoro F. (2019). Inkjet-printed PEDOT:PSS multi-electrode arrays for low-cost: In vitro electrophysiology. Lab Chip.

[24] Bachmann B., Adly N.Y., Schnitker J., Yakushenko A., Rinklin P., Offenhäusser A., Wolfrum B. (2017). All-inkjet-printed gold microelectrode arrays for extracellular recording of action potentials. Flex. Print. Electron..

[25] Adly N., Weidlich S., Seyock S., Brings F., Yakushenko A., Offenhäusser A., Wolfrum B. (2018). Printed microelectrode arrays on soft materials: From PDMS to hydrogels. npj Flex. Electron..

[26] Tonello S., Borghetti M., Lopomo N.F., Serpelloni M., Sardini E., Marziano M., Serzanti M., Uberti D., DELL’ERA P., Inverardi N. (2019). Ink-jet printed stretchable sensors for cell monitoring under mechanical stimuli: A feasibility study. J. Mech. Med. Biol..

[27] Plotczyk M., Higgins C.A. (2019). Skin biology. Biomaterials for Skin Repair and Regeneration.

[28] Boukamp P., Petrussevska R.I., Breitkreutz D., Hornung J., Markham A., Fusening N.E. (1988). Normal keratinization in a spontaneously immortalized aneuploid human keratinocyte cell line. J. Cell Biol..

[29] Martin P. (1997). Wound healing—Aiming for perfect skin regeneration. Science.

[30] Magdassi S., Grouchko M., Berezin O., Kamyshny A. (2010). Triggering the sintering of silver nanoparticles at room temperature. ACS Nano.

[31] Grouchko M., Kamyshny A., Mihailescu C.F., Anghel D.F., Magdassi S. (2011). Conductive inks with a ‘built-in’ mechanism that enables sintering at room temperature. ACS Nano.

[32] Han D.-W., Woo Y.I., Lee M.H., Lee J.H., Lee J., Park J. (2012). In-vivo and in-vitro biocompatibility evaluations of silver nanoparticles with antimicrobial activity. J. Nanosci. Nanotechnol..

[33] Greulich C., Kittler S., Epple M., Muhr G., Köller M. (2009). Studies on the biocompatibility and the interaction of silver nanoparticles with human mesenchymal stem cells (hMSCs). Langenbeck’s Arch. Surg..

[34] Pauksch L., Hartmann S., Rohnke M., Szalay G., Alt V., Schnettler R., Lips K.S. (2014). Biocompatibility of silver nanoparticles and silver ions in primary human mesenchymal stem cells and osteoblasts. Acta Biomater..

[35] Nemani K.V., Moodie K.L., Brennick J.B., Su A., Gimi B. (2013). In vitro and in vivo evaluation of SU-8 biocompatibility. Mater. Sci. Eng. C Mater. Biol. Appl..

[36] Solly K., Wang X., Xu X., Strulovici B., Zheng W. (2004). Application of real-time cell electronic sensing (RT-CES) technology to cell-based assays. Assay Drug Dev. Technol..

[37] Vedula S.R.K., Ravasio A., Lim C.T., Ladou B. (2013). Collective cell migration: A mechanistic perspective. Physiology.

[38] Riahi R., Yang Y., Zhang D.D., Wong P.K. (2012). Advances in wound-healing assays for probing collective cell migration. J. Lab. Autom..

[39] Teixeira A.I., Abrams G.A., Bertics P.J., Murphy C.J., Nealey P.F. (2003). Epithelial contact guidance on well-defined micro- and nanostructured substrates. J. Cell Sci..

[40] Pennacchio F.A., Garma L.D., Matino L., Santoro F. (2018). Bioelectronics goes 3D: New trends in cell-chip interface engineering. J. Mater. Chem. B.

[41] De Araujo A.L.A., Claudel J., Kourtiche D., Nadi M. (2019). Use of an insulation layer on the connection tracks of a biosensor with coplanar electrodes to increase the normalized impedance variation. Biosensors.

[42] Schwan H.P. (1963). Electric characteristics of tissues. Biophysik.

[43] Caviglia C., Zor K., Canepa S., Carminati M.A.R.C.O., Larsen L.B., Raiteri R., Andresen T.L., Heiskanen A., Emnéus J. (2015). Interdependence of initial cell density, drug concentration and exposure time revealed by real-time impedance spectroscopic cytotoxicity assay. Analyst.

[44] Cui Y., An Y., Jin T., Zhang F., He P. (2017). Real-time monitoring of skin wound healing on nano-grooves topography using electric cell-substrate impedance sensing (ECIS). Sens. Actuators B Chem..

[45] Patel P., Markx G.H. (2008). Dielectric measurement of cell death. Enzyme Microb. Technol..

[46] Mazlan N.S., Ramli M.M., Abdullah M.M.A.B., Halin D.C., Isa S.M., Talip L.F.A., Danial N.S., Murad S.Z. (2017). Interdigitated electrodes as impedance and capacitance biosensors: A review. AIP Conf. Proc..

[47] Igreja R., Dias C.J. (2004). Analytical evaluation of the interdigital electrodes capacitance for a multi-layered structure. Sens. Actuators A Phys..

[48] Igreja R., Dias C.J. (2011). Extension to the analytical model of the interdigital electrodes capacitance for a multi-layered structure. Sens. Actuators A Phys..

[49] Grimnes S., Martinsen Ø.G. (2015). Chapter 9—Data and Models.

[50] Someya T., Bao Z., Malliaras G.G. (2016). The rise of plastic bioelectronics. Nature.

[51] Derby B. (2010). Inkjet printing of functional and structural materials: Fluid property requirements, feature stability, and resolution. Annu. Rev. Mater. Res..

[52] Sele C.W., von Werne T., Friend R.H., Sirringhaus H. (2005). Lithography-free, self-aligned inkjet printing with sub-hundred-nanometer resolution. Adv. Mater..

[53] Farooqui M.F., Shamim A. (2016). Low Cost Inkjet Printed Smart Bandage for Wireless Monitoring of Chronic Wounds. Sci. Rep..

[54] Sandison M.E., Anicet N., Glidle A., Cooper J.M. (2002). Optimization of the geometry and porosity of microelectrode arrays for sensor design. Anal. Chem..

[55] Min J., Baeumner A.J. (2004). Characterization and optimization of interdigitated ultramicroelectrode arrays as electrochemical biosensor transducers. Electroanalysis.

[56] Pejcic B., de Marco R. (2006). Impedance spectroscopy: Over 35 years of electrochemical sensor optimization. Electrochim. Acta.

[57] Radke S.M., Alocilja E.C. (2004). Design and fabrication of a microimpedance biosensor for bacterial detection. IEEE Sens. J..

[58] Price D.T., Rahman A.R.A., Bhansali S. (2009). Design rule for optimization of microelectrodes used in electric cell-substrate impedance sensing (ECIS). Biosens. Bioelectron..

[59] Koley D., Bard A.J. (2010). Triton X-100 concentration effects on membrane permeability of a single HeLa cell by scanning electrochemical microscopy (SECM). Proc. Natl. Acad. Sci. USA.

